# Supplementary material for: Spousal concordance in telomere length: New evidence from older adults in the US
Source: PLoS One. 2018 Nov 1;13(11):e0202388. doi: 10.1371/journal.pone.0202388 (PMC6211628; doi:10.1371/journal.pone.0202388)
Supplement: S1 Table — Notes: observations with Telomere Length above 3 are not included. (DOCX) [file pone.0202388.s001.docx]

S1 Table

Descriptive Statistics of Full Sample and Pooled Spousal Sample

| Variable | Obs | Mean | Std Dev | Min | Max |
| --- | --- | --- | --- | --- | --- |
| Telomere Length | 5733 | 1.32 | 0.33 | 0.20 | 2.98 |
| Female Indicator | 5733 | 0.59 | 0.49 | 0.00 | 1.00 |
| Age (2008) | 5733 | 68.77 | 10.34 | 26.00 | 107.00 |
| Educational Attainment | 5725 | 12.54 | 3.19 | 0.00 | 17.00 |
| White | 5733 | 0.82 | 0.39 | 0.00 | 1.00 |
| Black | 5733 | 0.13 | 0.34 | 0.00 | 1.00 |
| Other Race | 5733 | 0.05 | 0.22 | 0.00 | 1.00 |
| Number of Marriages | 5721 | 1.40 | 0.77 | 0.00 | 7.00 |
| Single Marriage | 5733 | 0.65 | 0.48 | 0.00 | 1.00 |
|  |  |  |  |  |  |
| Variable | Obs | Mean | Std Dev | Min | Max |
| Telomere Length | 3022 | 1.32 | 0.33 | 0.23 | 2.96 |
| Female Indicator | 3022 | 0.50 | 0.50 | 0.00 | 1.00 |
| Age (2008) | 3022 | 67.09 | 9.74 | 26.00 | 93.00 |
| Educational Attainment | 3019 | 12.79 | 3.17 | 0.00 | 17.00 |
| White | 3022 | 0.86 | 0.35 | 0.00 | 1.00 |
| Black | 3022 | 0.09 | 0.28 | 0.00 | 1.00 |
| Other Race | 3022 | 0.05 | 0.23 | 0.00 | 1.00 |
| Number of Marriages | 3021 | 1.45 | 0.76 | 0.00 | 7.00 |
| Single Marriage | 3022 | 0.66 | 0.47 | 0.00 | 1.00 |

Notes: observations with Telomere Length above 3 are not included.
