# Supplementary material for: Spousal concordance in telomere length: New evidence from older adults in the US
Source: PLoS One. 2018 Nov 1;13(11):e0202388. doi: 10.1371/journal.pone.0202388 (PMC6211628; doi:10.1371/journal.pone.0202388)
Supplement: S2 Table — Notes: Standard errors in parentheses. *** p<0.01, ** p<0.05, * p<0.1. (DOCX) [file pone.0202388.s002.docx]

S2 Table

Associations between Individual Characteristics and Telomere Length in the HRS

|  | TL | TL | TL | TL |
| --- | --- | --- | --- | --- |
| Sample | Full | Spousal | Wives | Husbands |
|  |  |  |  |  |
| Female Indicator | 0.028*** | 0.042*** |  |  |
|  | (0.009) | (0.012) |  |  |
| Age (2008) | -0.005*** | -0.005*** | -0.006*** | -0.005*** |
|  | (0.000) | (0.001) | (0.001) | (0.001) |
| Educational Attainment | 0.001 | 0.001 | 0.001 | 0.001 |
|  | (0.001) | (0.002) | (0.003) | (0.003) |
| Black Race | 0.094*** | 0.108*** | 0.113*** | 0.104*** |
|  | (0.013) | (0.021) | (0.030) | (0.030) |
| Other Race | 0.023 | 0.040 | 0.034 | 0.047 |
|  | (0.020) | (0.026) | (0.036) | (0.039) |
| Number of Marriages | -0.019*** | -0.008 | -0.009 | -0.007 |
|  | (0.006) | (0.008) | (0.011) | (0.011) |
| Constant | 1.631*** | 1.645*** | 1.725*** | 1.604*** |
|  | (0.039) | (0.053) | (0.073) | (0.075) |
|  |  |  |  |  |
| Observations | 5,715 | 3,019 | 1,508 | 1,508 |
| R-squared | 0.038 | 0.044 | 0.044 | 0.030 |

Standard errors in parentheses. *** p<0.01, ** p<0.05, * p<0.1
