# Supplementary material for: Spousal concordance in telomere length: New evidence from older adults in the US
Source: PLoS One. 2018 Nov 1;13(11):e0202388. doi: 10.1371/journal.pone.0202388 (PMC6211628; doi:10.1371/journal.pone.0202388)
Supplement: S3 Table — Notes: 1 SD + M is one standard deviation more than the mean. Standard errors in parentheses. *** p<0.01, ** p<0.05, * p<0.1. (DOCX) [file pone.0202388.s003.docx]

S3 Table

Adjusted Associations between Spousal Telomere Length in the HRS: Exploring Robustness to Removal of Outlier TL Measurements

| Outcome | TL | TL | TL | TL | TL | TL | TL | TL | TL | TL | TL |
| --- | --- | --- | --- | --- | --- | --- | --- | --- | --- | --- | --- |
| Sample | Spouses | Spouses | Spouses | Spouses | Spouses | Spouses | Spouses | Spouses | Spouses | Spouses | Spouses |
| Exclude | >3 TL | >1 SD +M | >2 SD +M | >3 SD +M | >4 SD +M | >5 SD +M | >6 SD +M | >7 SD +M | >8 SD +M | >9 SD +M | >10 SD +M |
|  |  |  |  |  |  |  |  |  |  |  |  |
| Spousal TL | 0.085*** | 0.086*** | 0.102*** | 0.088*** | 0.073*** | 0.087*** | 0.114*** | 0.095*** | 0.095*** | 0.095*** | 0.095*** |
|  | (0.027) | (0.030) | (0.027) | (0.027) | (0.027) | (0.027) | (0.028) | (0.028) | (0.028) | (0.028) | (0.028) |
| Husband Education | -0.001 | -0.001 | -0.003 | -0.003 | -0.003 | -0.001 | 0.000 | -0.001 | -0.001 | -0.001 | -0.001 |
|  | (0.003) | (0.003) | (0.003) | (0.003) | (0.003) | (0.003) | (0.003) | (0.003) | (0.003) | (0.003) | (0.003) |
| Husband Black Race | 0.094 | -0.100 | -0.011 | -0.028 | -0.016 | 0.093 | 0.089 | 0.087 | 0.087 | 0.087 | 0.087 |
|  | (0.082) | (0.067) | (0.073) | (0.079) | (0.084) | (0.083) | (0.087) | (0.090) | (0.090) | (0.090) | (0.090) |
| Husband Other Race | 0.042 | 0.039 | 0.009 | 0.029 | 0.060 | 0.041 | 0.039 | 0.035 | 0.035 | 0.035 | 0.035 |
|  | (0.045) | (0.036) | (0.039) | (0.042) | (0.044) | (0.045) | (0.048) | (0.049) | (0.049) | (0.049) | (0.049) |
| Husband Single Marriage | 0.020 | 0.006 | 0.021 | 0.015 | 0.009 | 0.021 | 0.022 | 0.022 | 0.022 | 0.022 | 0.022 |
|  | (0.025) | (0.020) | (0.022) | (0.023) | (0.024) | (0.025) | (0.027) | (0.027) | (0.027) | (0.027) | (0.027) |
| Wife Education | 0.005 | 0.005* | 0.007** | 0.006* | 0.008** | 0.005 | 0.004 | 0.005 | 0.005 | 0.005 | 0.005 |
|  | (0.004) | (0.003) | (0.003) | (0.003) | (0.003) | (0.004) | (0.004) | (0.004) | (0.004) | (0.004) | (0.004) |
| Wife Black Race | -0.006 | 0.093 | 0.037 | 0.070 | 0.081 | -0.005 | -0.009 | -0.009 | -0.009 | -0.009 | -0.009 |
|  | (0.084) | (0.068) | (0.075) | (0.080) | (0.086) | (0.085) | (0.089) | (0.092) | (0.092) | (0.092) | (0.092) |
| Wife Other Race | 0.006 | -0.013 | 0.021 | -0.005 | -0.007 | 0.005 | 0.003 | -0.002 | -0.002 | -0.002 | -0.002 |
|  | (0.043) | (0.035) | (0.037) | (0.040) | (0.042) | (0.043) | (0.046) | (0.047) | (0.047) | (0.047) | (0.047) |
| Wife Single Marriage | -0.005 | -0.021 | -0.018 | -0.014 | -0.005 | -0.004 | 0.003 | -0.007 | -0.007 | -0.007 | -0.007 |
|  | (0.025) | (0.019) | (0.021) | (0.023) | (0.024) | (0.025) | (0.026) | (0.027) | (0.027) | (0.027) | (0.027) |
| Husband Age | -0.004** | -0.002* | -0.004*** | -0.004*** | -0.004** | -0.004** | -0.004** | -0.003* | -0.003* | -0.003* | -0.003* |
|  | (0.002) | (0.001) | (0.001) | (0.002) | (0.002) | (0.002) | (0.002) | (0.002) | (0.002) | (0.002) | (0.002) |
| Wife Age | -0.000 | -0.000 | 0.000 | 0.000 | 0.000 | -0.000 | 0.001 | -0.000 | -0.000 | -0.000 | -0.000 |
|  | (0.002) | (0.001) | (0.001) | (0.001) | (0.002) | (0.002) | (0.002) | (0.002) | (0.002) | (0.002) | (0.002) |
| Constant | 1.414*** | 1.217*** | 1.316*** | 1.402*** | 1.398*** | 1.410*** | 1.310*** | 1.338*** | 1.338*** | 1.338*** | 1.338*** |
|  | (0.091) | (0.078) | (0.080) | (0.085) | (0.088) | (0.092) | (0.096) | (0.098) | (0.098) | (0.098) | (0.098) |
| Observations | 1,492 | 1,176 | 1,394 | 1,460 | 1,483 | 1,493 | 1,498 | 1,503 | 1,503 | 1,503 | 1,503 |
| R-squared | 0.038 | 0.030 | 0.038 | 0.036 | 0.033 | 0.037 | 0.033 | 0.028 | 0.028 | 0.028 | 0.028 |

Notes: 1 SD + M is one standard deviation more than the mean. Standard errors in parentheses. *** p<0.01, ** p<0.05, * p<0.1
